# Using the 12-item short form health survey (SF-12) to assess self rated health of an engaged population impacted by hurricane Harvey, Houston, TX

**DOI:** 10.1186/s12889-020-8349-x

**Published:** 2020-02-19

**Authors:** Garett T. Sansom, Katie Kirsch, Jennifer A. Horney

**Affiliations:** 1 Research Assistant Professor, Environmental and Occupational Health, Texas A&M University School of Public Health, College Station, TX 77840 USA; 2 Research Associate, Epidemiology and Biostatistics, Texas A&M University School of Public Health, College Station, TX 77840 USA; 3 Professor and Founding Director, Epidemiology Program, University of Delaware, Newark, DE 19713 USA

**Keywords:** Self-rated health, Disasters, Resilience, Social capital, Community engagement

## Abstract

**Background:**

In the last decade there has been an increase in community-based organizations providing support and educational outreach to populations effected by hazards. Prior research has demonstrated various roles that community social capital can play in both the enhancement of disaster preparedness and the mitigation of physical and mental health impacts following a natural disaster.

**Methods:**

To assess self-reported health of residents of South Houston, Texas impacted by Hurricane Harvey, attendees of a community event completed a survey that included the 12 item short form health survey version 2 (SF-12v2).

**Results:**

Although survey participants were older and more likely to be African-American than the overall population of Houston, they had higher mental health composite scores that the national average, with increases in mental wellbeing associated with a longer length of residence in their neighborhood.

**Conclusions:**

The City of Houston, with highly segregated, socially vulnerable populations at high risk from natural hazards, should consider ways to support community engagement around disaster preparedness, response, and recovery that may build community cohesion and improve post-disaster mental health.

## Background

Research has consistently demonstrated that environmental justice communities are disproportionately impacted by proximate sources of pollution, toxic exposures, and other hazards that result in excess risks for human health outcomes based on race, income, housing segregation, and other factors [1–5]. However, individuals living in communities at risk may experience environmental issues in different ways. For example, in a previous study of predominantly Hispanic residents of an environmental justice community located adjacent to the Houston Ship Channel (HSC), respondents reported significantly lower self-reported physical health (*p* < 0.001), which was negatively correlated with the length of time a resident had been living in the neighborhood (*p* < 0.001) [6]. However, among this population, mental health scores did not differ from the national average nor change as a function of time living in the neighborhood. In a study of residents of Texas Gulf Coast counties conducted after Hurricane Harvey, residents exposed to Harvey had lower self-rated mental health than the U.S national population (mean mental composite score (MCS) = 34.58, standard deviation (SD) = 8.89) [7]. Because the 12-item Short Form Health Survey version 2 (SF-12v2) is a widely validated measure of self-reported physical and mental health [8], and has been infrequently used to assess environmental or natural disaster related exposures, this study attempted to characterize the self-reported mental and physical health of members of a socially and physically vulnerable South Houston region who were highly engaged in a community non-profit whose focus was related to emergency preparedness and recovery following Hurricane Harvey. Specifically, identifying the potential mitigating effects of increased engagement on the mental health of communities that experience natural disasters.

Environmental justice communities are communities with low socioeconomic status (SES) and minority-majority populations that experience disproportionate exposure to negative environmental conditions compared to more affluent, majority white communities in the U.S. [9, 10]. Over the past 50 years, race has remained one of the most important factors in explaining residential segregation in the City of Houston [11]. Industrial development has primarily been concentrated in east and south Houston neighborhoods, while more affluent residents and the services and amenities associated with them, have primarily developed in the western and northern portions of Houston [12]. Among other factors, these patterns of development mean that African-American, Hispanic, and other minority residents of Houston became increasingly integrated with one another in east and south Houston [13].

A lack of zoning in Houston has furthered these types of land use and urban development inequities, while exposing residents to pollution from more freeway miles than any comparable region of the U.S. [14, 15]. Vulnerable communities in the City of Houston are disproportionately exposed to polycyclic aromatic hydrocarbons (PAHs) associated with transportation infrastructure [16, 17], heavy metals in standing water [18], detectable lead levels in drinking water [19], and outdated and ineffective infrastructure to handle flooding events [20]. These conditions are being further exacerbated by simultaneous increases in the severity and frequency of inland precipitation [21], the combined effects of sea-level rise, subsidence, and storm surge [22], and increases in the proportion of impermeable surfaces as the result of population growth and development [23].

This increased flood risk in Houston was evident following Hurricane Harvey, which made landfall along the Texas Gulf Coast in August 2017 and became the wettest tropical cyclone to impact the U.S., inundating 70% of the City of Houston at a level of at least 18 in [24].. Using inundation maps produced by the Federal Emergency Management Agency (FEMA) and the Harris County Flood Control District (HCFCD) following Hurricane Harvey, Chakraborty, Collins, and Grineski (2019a) demonstrated that the greatest floodwater inundation levels were observed in areas with greater proportions of non-Hispanic Black and economically disadvantaged residents [25]. The extent of flooding, as measured by FEMA’s Hurricane Harvey Inundation Footprint aerial map, was statistically significantly higher in neighborhoods with a higher proportion of disabled residents, with the greatest risk observed among ambulatory- and cognitive-type disabilities [26]. Across Harris County, neighborhoods with a higher proportion of individuals with one or more disabilities, including neighborhoods with high rates of cognitive- and hearing-type disabilities, were more likely to be located nearer to facilities required by the U.S. Environmental Protection Agency (EPA) to submit Risk Management Plans due to their hazardous potential [26].

## Methods

### Study setting

**Fig. 1:**
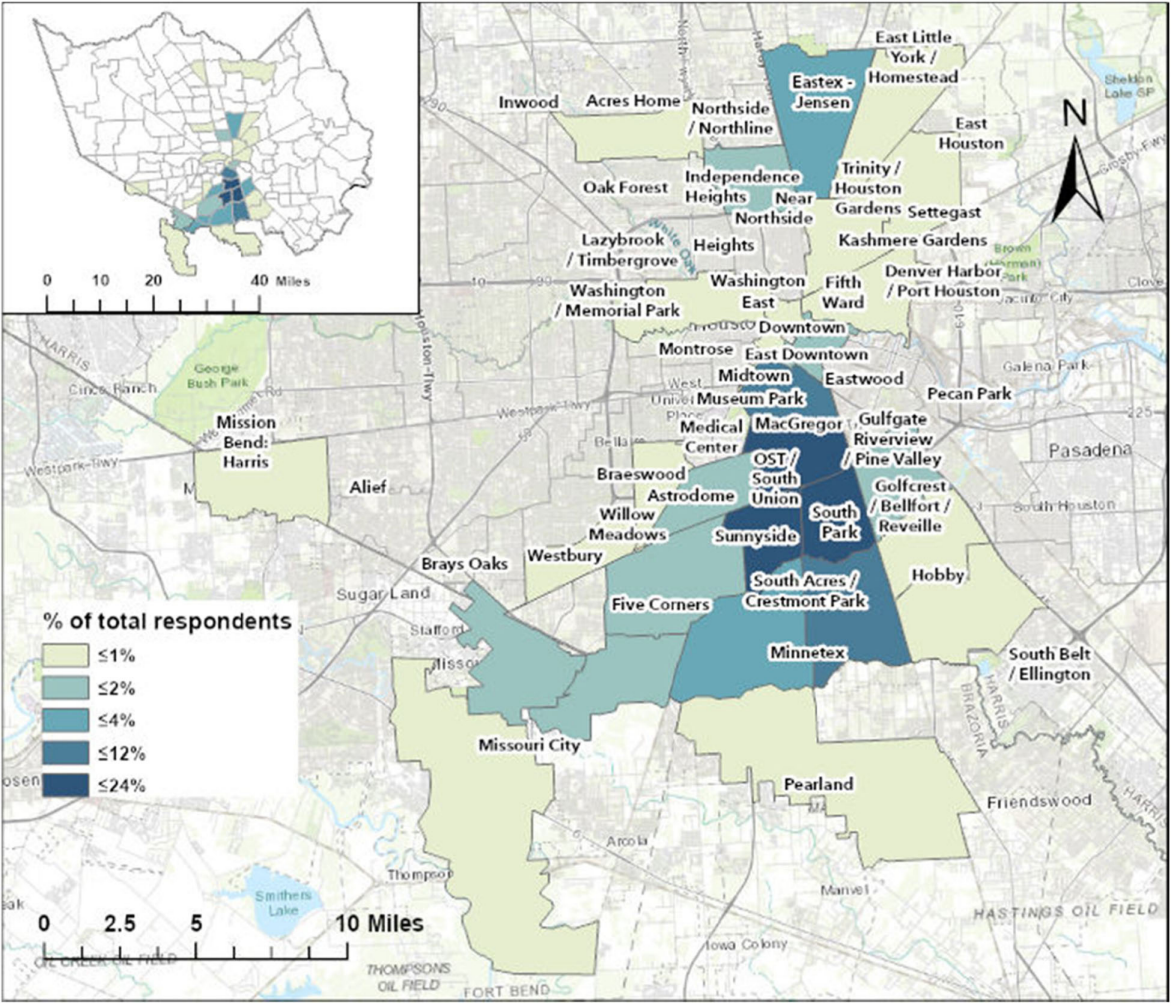
Residential Address of Survey Participants by Houston Super Neighborhood; Percent of Respondents (*N*= 153). This figure depicts the neighborhoods in which participants primary homes are located. This figure was created by the authors utilizing ArcGIS by ESRI

In October 2017, research and engagement staff from the Texas A&M University Institute for Sustainable Communities (IfSC) attended a Community Breakfast event hosted by Charity Productions, a Houston-based non-profit focused on emergency management and public safety issues. Charity Productions hosts quarterly breakfast meetings where community attendees can participate in educational outreach, civic engagement, and community strengthening activities. Often attended by more than 200 residents of Houston, the community breakfasts also include the distribution of fact-based information about hazards and disasters and the opportunity for attendees to participate in community-engaged research projects that focus on collecting data that can help increase community cohesion and resilience. During the October 2017 event, held approximately 2 months after Hurricane Harvey made landfall along the Texas Gulf Coast south of Houston, the agenda focused on how community agencies and residents could partner with academic institutions to better prepare for and recover from increasingly frequent and severe natural hazards like Hurricane Harvey. Attendees were those who had participated within Charity Productions events for several years and attended the quarterly meetings and periodic seminars. Following presentations by elected officials and academic researchers, attendees were asked to complete a survey created by IfSC. More than 90% (138 of 153) of those who attended the breakfast and completed the survey reported a residential address in south Houston (Fig. 1).

### Survey instrument

The survey included three sections. In the first part of the survey, respondents provided demographic information (e.g., gender, race/ethnicity, and age), location of primary residence, and years of tenure in current neighborhood. A second section was included to gauge respondent’s perceptions of environmental risk and included questions related to concerns about air pollution, flooding, proximity to industrial facilities, access to public transportation, and housing conditions. The final section of the survey included the SF-12v2, which was adapted from the medical outcomes study [27]. The SF-12v2 has been validated for use in predicting the mental and physical health of populations without targeting specific health outcomes and shown to be reliable in U.S. and international populations [28, 29]. The SF-12v2 has also been validated in several different languages [30, 31], within low SES populations [32], and among immigrant populations in the U.S. [33]. Using a norm-based algorithm it produces a composite score for self-reported mental and physical between 0 and 100, which allows for comparison between study populations and national averages [34].

### Data collection

Eligible participants included members of Charity Productions and community breakfast attendees aged 18 years and older whose primary residence was in the Greater Houston, Texas area. Following the presentations by elected officials and academic researchers about post-Harvey recovery progress, attendees were given a brief overview of the purpose of the survey. Trained engagement staff and graduate students distributed the surveys, confirmed eligibility, and collected completed surveys. Response rates were calculated by summing the total number of eligible attendees, removing academic guests and invited speakers from outside the community, and dividing this number by those who returned a completed survey.

### Data analysis

Responses to SF-12v2 questions were used to estimate a mental composite score (MCS) and physical composite score (PCS) for each subject on a 0- and 100- point scale in accordance with the methods outlined by Ware et al. (2000) [34]. The national mean scores for both mental and physical health are standardized at 50; scores above this represent higher, or healthier, individuals than average. Overall and gender stratified mean MCS and PCS values for respondents were calculated and compared with the national mean using two-tailed t-tests. Multiple linear regression was used to assess the impact of time spent in the neighborhood, age, and gender on MCS and PCS. Coefficients of the covariates, along with their corresponding 95% confidence intervals (95% CI) were reported. Two-way scatterplots were created to visually examine associations between MCS and PCS and tenure of residence.

The City of Houston is organized into 88 Super Neighborhoods, which are geographically defined areas where residents, civic groups, and businesses work together to plan and set priorities that address shared concerns [35]. The primary residence of survey respondents were categorized into three groups: 1) South Houston Super Neighborhoods including Sunnyside, Southpark, South Acres/Crestmont Park, Central Southwest, and Greater Hobby Area; 2) Other Houston Super Neighborhoods including Midtown, Downtown, Eastex, and Greater Inwood; and 3) Areas outside the City of Houston, including Brenham, Humble, and League City. Statistical analyses were conducted using STATA 15 (College Station, TX) and Microsoft Excel (Redmond, Washington).

## Results

One hundred fifty three surveys were completed at the October 2017 community breakfast (Response Rate = 81.4%). The majority of respondents were female (83.7%, *N* = 128), African-American (90.2%, *N* = 138), and 66 years of age or older (79.1%, *N* = 121), with the average age being 72 years (SD = 10.1 years) (Table 1). Ninety percent (N = 138) of respondents reported living in a Super Neighborhood located in South Houston (Fig. 1), and the average length of residence was 34.3 years (SD = 20.2 years). Two in five (39.1%; *N* = 61) participants reported that flooding was an issue in their neighborhood, and 35.9% (*N* = 56) reported pollution was their largest concern. Fifteen percent (*N* = 24) identified public transportation as an unmet need and 10.3% (*N* = 16) identified insufficient public services and city maintenance issues.

**Table 1:** Distribution of study respondents by gender, race/ethnicity, age, and location of primary residence

Characteristic	N (%)^**a**^
Gender	
Male	25 (16.3%)
Female	128 (83.7%)
Race / Ethnicity	
Non-Hispanic White	1 (0.7%)
African American	138 (90.2%)
Refused	14 (9.2%)
Age in Years	
18–35	2 (1.3%)
36–65	30 (19.6%)
66–80	80 (52.3%)
80+	27 (17.6%)
Refused	14 (9.2%)
Location of Primary Residence	
South Houston, Texas Super Neighborhoods	138 (90.2%)
Other Houston, Texas Super Neighborhoods	8 (5.2%)
Areas outside of Houston, Texas city limits	6 (3.9%)
Refused	1 (0.7%)

^a^Values may not equal 100% due to rounding

**Fig. 2:**
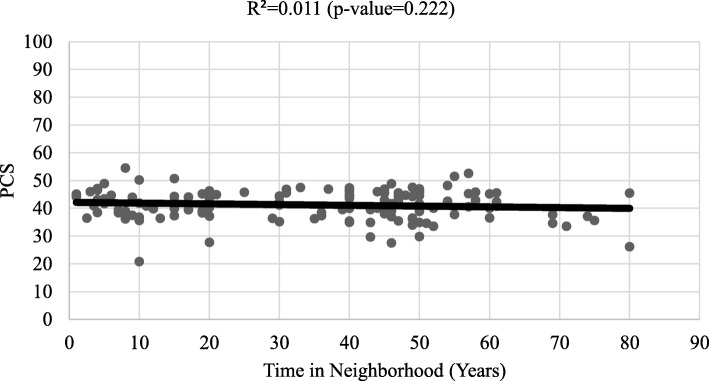
Physical composite score (MCS) by years lived in the neighborhood

**Fig. 3:**
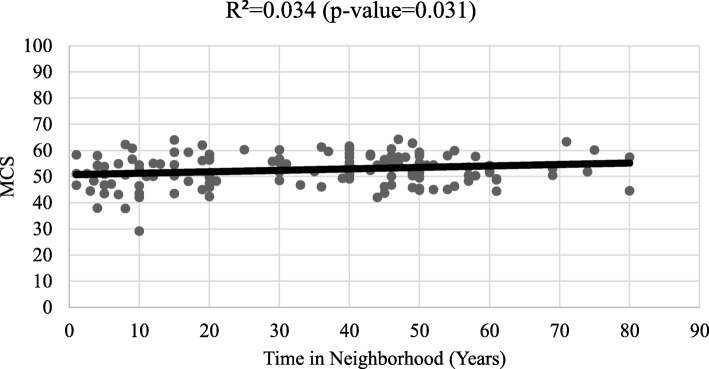
Mental composite score (MCS) by years lived in the neighborhood

Respondents had higher MCS than the U.S. mean of 50, with MCS for women of 52.19 (95% CI: 51.06, 53.32) and MCS for men of 53.22 (95% CI: 50.87, 55.56). However, PCS for women was 41.59 (95% CI: 40.74, 42.45) and PCS for men was 40.15 (95% CI: 36.94, 43.47) significantly lower - nearly a full standard deviation - below national mean scores (Table 2). These findings remained consistent after adjusting for the age of respondents (There was no statistically significant correlation between PCS and years lived in the neighborhood (Fig. 2). However, longer length of residence in the neighborhood was positively correlated with MCS (R^2^ = 0.034; p = 0.031) (Fig. 3).

**Table 2:** Two-tailed *t* tests of mean mental and physical composite scores by gender compared to the standardized national average of 50

Group	*t* value	Mean	95% CI	*p*-value
Mental Composite Score				
Male	2.83	53.22	50.87, 55.56	0.005
Female	3.83	52.19	51.06, 53.32	< 0.001
Physical Composite Score				
Male	−6.32	40.15	36.94, 43.37	< 0.001
Female	−19.53	41.59	40.74, 42.45	< 0.001

Although mean MCS and PCS among both male and female respondents were significantly different than the standardized national average, in multiple linear regression models, other than residence tenure and MCS scores, there were no differences in MCS or PCS by gender or age of respondents and PCS was not affected by tenure (Table 3).

**Table 3:** Multiple linear regression comparing mental and physical composite scores by gender, tenure of current residence, and age

Group	Coef	Std Err.	95% CI	*p*-value
Mental Composite Score				
Gender (Female)	− 1.24	1.46	−4.13, 1.66	0.394
Tenure of Residence	0.06	0.03	0.01, 0.12	0.031
Age	0.07	0.06	−0.53, 0.19	0.282
Physical Composite Score				
Gender (Female)	0.48	1.28	−2.06, 3.03	0.711
Tenure of Residence	−0.03	0.03	−0.08, − 0.02	0.211
Age	0.01	0.05	−0.09, 0.12	0.863

## Discussion

In this sample of Houston residents that attend communal events through Charity Productions and attended a community engagement event specifically focused on disaster preparedness and recovery from Hurricane Harvey, 90% of respondents reported living in a majority minority South Houston Super Neighborhood, including Central Southwest, Sunnyside, Southpark, South Acres / Crestmont Park, and Greater Hobby Area. South Houston neighborhoods shoulder a burden of excess rates of poverty and unemployment [36] and are susceptible to flood-related damage and exposure opportunities [37]. These neighborhoods have been identified as highly socially vulnerable and having low capacity to prepare for and respond to the stress of hazardous events due in part to their low SES, the prevalence of households that include children, the elderly, or disabled members, a lack of access to transportation, low rates of home ownership, and minority race/ethnicity [38].

While mental and physical health are generally correlated, in this sample respondents reported better than average MCS and worse than average PCS. This is noteworthy as previous research with similar populations has found a rough parity between MCS and PCS. For instance, a cross-sectional study of low-income African American residents of Nashville, Tennessee (*N* = 1721) utilized the SF-12v2 to assess mental and physical health, finding that mental health and physical health often rose or fell together and mean MCS and PCS were only 6 points apart [39]. In this study, there was more than a 12-point difference in respondent’s mean MCS and PCS, more than a standard deviation. These differences may offer insight into the benefits of promoting mental health among vulnerable communities through engagement in civic events focused on disaster preparedness, response, and recovery.

As this study was conducted among a group of community-engaged individuals attending one of several community events throughout the year, it is possible that their engagement was beneficial to their mental health resilience to the impacts of Hurricane Harvey. In research after Hurricane Sandy, Lowe et al. (2015) identified socioeconomic disadvantage, non-Hispanic Black race, and older age as characteristics associated with higher rates of posttraumatic stress [40]. After Hurricane Katrina, white women, older women, and women with a partner were found to be more resilient from depression and post-traumatic stress disorder [41]. However, several models emerged after the Hurricane and the Deep Water Horizon oil spill that demonstrated ways in which faith-based, governmental, and academic groups could come together to improve disaster mental health and resilience [42, 43]. These resident’s long-term tenure in their neighborhoods may have facilitated community connections – of which attendance at this community event was one manifestation – that were protective and supported resilience that was operationalized through a relatively rapid return to a pre-disaster state [44]. Further, increased social cohesion, even outside experiencing hazardous events, has been demonstrated to mitigate many potentially negative impacts on mental health and could account for the relatively high MCS scores [45].

Also, among this sample, physical health remained consistent over increasing community tenure, whereas mental health showed a marked and significant improvement the longer individuals lived within their respective communities. Ample evidence exists indicating physical health issues within this community, for instance the mean life expectancy for Harris County and Houston, Texas in 2014 was approximately 79 years, while Sunnyside residents had an estimated mean life expectancy of only 71 years from 2010 to 2015 [46]. Although our sample was small, this lends support to the hypothesis that community cohesion can improve mental health, even in an aging community with physical health issues and provides initial evidence for a pathway to improve lives through interventions targeting communal networking improvement projects.

This study has several important limitations. Primarily this study lacks a matched comparison group to directly assess our results with other groups of differing SES, community cohesion, and exposure to the hurricane. Secondly, since the survey respondents were all attendees at a community engagement event, these findings may not be generalizable to residents of the same Super Neighborhoods that are not engaged in similar organizations. Further, as this event required individuals to travel to a designated location, it may have missed the most vulnerable and those with the greatest physical or mental health challenges. Although no mental and physical health assessments involving this population were conducted before Hurricane Harvey, the present study can provide baseline measures with which to compare future research and generate improved hypotheses. A strength of this study is that the survey was self-administered, limiting potential response bias compared to an interviewer administered survey [47].

## Conclusion

Improving our understanding of the complex relationship between the mental and physical health impacts of disasters and the potential for active engagement to mitigate these impacts through increasing individual resilience, even among minority residents of environmental justice communities, will require additional longitudinal research. In the meantime, these data can serve as a baseline for understanding the potential benefits of engagement on mental wellbeing after natural disasters within vulnerable communities.

## Data Availability

The datasets used and/or analyzed during the current study are available from the corresponding author on reasonable request.

## Abbreviations

EPA: U.S. environmental protection agency
FEMA: Federal emergency management agency
HCFCD: Harris county flood control district
HSC: Houston ship channel
IfSC: The Texas A&M university institute for sustainable communities
MCS: Mental composite score
PAH: Polycyclic aromatic hydrocarbons
PCS: Physical composite score
SES: Low socioeconomic status
SF-12v2: 12 item short form health survey version 2
